# Regulation of Exocytotic Fusion Pores by SNARE Protein Transmembrane Domains

**DOI:** 10.3389/fnmol.2017.00315

**Published:** 2017-10-10

**Authors:** Zhenyong Wu, Sathish Thiyagarajan, Ben O’Shaughnessy, Erdem Karatekin

**Affiliations:** ^1^Department of Cellular and Molecular Physiology, School of Medicine, Yale University, New Haven, CT, United States; ^2^Nanobiology Institute, Yale University, West Haven, CT, United States; ^3^Department of Physics, Columbia University, New York, NY, United States; ^4^Department of Chemical Engineering, Columbia University, New York, NY, United States; ^5^Department of Molecular Biophysics and Biochemistry, Yale University, New Haven, CT, United States; ^6^Laboratoire de Neurophotonique, Université Paris Descartes, Faculté des Sciences Fondamentales et Biomédicales, Centre National de la Recherche Scientifique (CNRS), Paris, France

**Keywords:** exocytosis, SNARE, transmembrane domain, fusion pore, membrane fusion

## Abstract

Calcium-triggered exocytotic release of neurotransmitters and hormones from neurons and neuroendocrine cells underlies neuronal communication, motor activity and endocrine functions. The core of the neuronal exocytotic machinery is composed of *soluble N-ethyl maleimide sensitive factor attachment protein receptors* (SNAREs). Formation of complexes between vesicle-attached v- and plasma-membrane anchored t-SNAREs in a highly regulated fashion brings the membranes into close apposition. Small, soluble proteins called Complexins (Cpx) and calcium-sensing Synaptotagmins cooperate to block fusion at low resting calcium concentrations, but trigger release upon calcium increase. A growing body of evidence suggests that the transmembrane domains (TMDs) of SNARE proteins play important roles in regulating the processes of fusion and release, but the mechanisms involved are only starting to be uncovered. Here we review recent evidence that SNARE TMDs exert influence by regulating the dynamics of the fusion pore, the initial aqueous connection between the vesicular lumen and the extracellular space. Even after the fusion pore is established, hormone release by neuroendocrine cells is tightly controlled, and the same may be true of neurotransmitter release by neurons. The dynamics of the fusion pore can regulate the kinetics of cargo release and the net amount released, and can determine the mode of vesicle recycling. Manipulations of SNARE TMDs were found to affect fusion pore properties profoundly, both during exocytosis and in biochemical reconstitutions. To explain these effects, TMD flexibility, and interactions among TMDs or between TMDs and lipids have been invoked. Exocytosis has provided the best setting in which to unravel the underlying mechanisms, being unique among membrane fusion reactions in that single fusion pores can be probed using high-resolution methods. An important role will likely be played by methods that can probe single fusion pores in a biochemically defined setting which have recently become available. Finally, computer simulations are valuable mechanistic tools because they have the power to access small length scales and very short times that are experimentally inaccessible.

## Introduction

Coordinated neuronal communication and motor activity rely on tightly controlled release of neurotransmitters. Secretion of hormones is likewise finely tuned, since these compounds control the physiological activities of organs and cells. Both neurotransmitters and hormones are packaged into intracellular secretory vesicles (synaptic vesicles or secretory granules, respectively) and are secreted via calcium-triggered exocytosis. Exocytosis is a multi-step process, involving translocation of secretory vesicles to release sites at the plasma membrane, maturation (called “priming”) to a state of fusion-readiness, and opening of a fusion pore in response to an increase in the local calcium concentration (Sudhof and Rothman, 2009; Jahn and Fasshauer, 2012; Rizo and Xu, 2015).

The late stages of exocytosis (from maturation at the fusion site to pore dilation) involve about a dozen proteins, many of which are essential. Munc13 is a large priming factor that cooperates with Munc18 to direct SNARE assembly (Rizo and Xu, 2015; Baker and Hughson, 2016). Synaptotagmin-1 (Syt1) and Complexin (Cpx) cooperate to inhibit fusion at resting (low) calcium and to induce rapid fusion upon a rise in calcium (Chapman, 2008; Diao et al., 2012; Rizo and Xu, 2015; Lai et al., 2016). The fusion step itself requires formation of *trans* complexes between vesicular v- and plasma (target) membrane t-SNAREs that bridge the two membranes (Sudhof and Rothman, 2009). Syt and Cpx may contribute to pore creation (Martens et al., 2007; Hui et al., 2009; Kyoung et al., 2011; Brunger et al., 2015), as Syt couples calcium binding to fusion (Rizo and Xu, 2015) and Cpx somehow increases the efficiency of this process (Lai et al., 2016).

**Figure 1:**
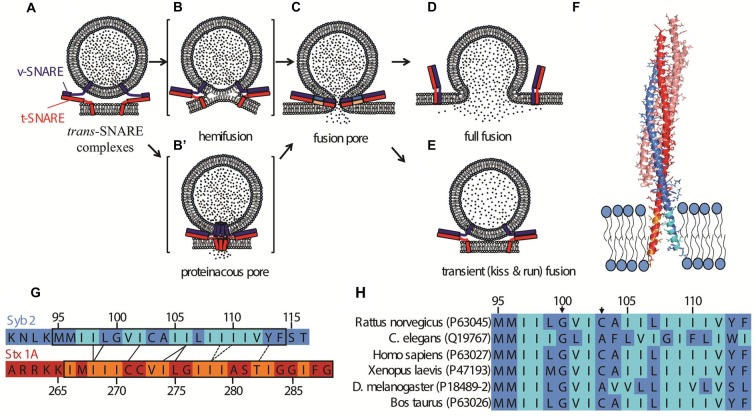
Possible fusion pathways for soluble N-ethyl maleimide sensitive factor attachment protein receptors (SNARE)-mediated fusion, and the structure of the post-fusion *cis* SNARE complex (Stein et al., 2009). **(A)** A synaptic vesicle is docked at the plasma membrane by *trans*-SNARE complexes. The exocytotic/neuronal v-SNARE Syb2 (blue) and the t-SNARE (Stx1 and SNAP25, depicted together in red) are anchored to the synaptic vesicle and the plasma membrane, respectively via their transmembrane domains (TMDs). Further zippering, coupled with the action of the calcium sensor Synaptotagmin-1 (not shown) and possibly other factors leads to the opening of a fusion pore **(C)**. Possible intermediate structures on the pathway to opening of the fusion pore include a hemifusion state **(B)** wherein the proximal leaflets, but not the distal ones, are fused. An alternative intermediate is a channel-like structure formed by oligomerization of the TMDs of soluble N-ethyl maleimide sensitive factor attachment protein receptors (SNAREs) in both membranes **(B′)**. Hetero-oligomerization of SNAREs with other proteins may also contribute to the channel structure. Expansion of the proteinaceous pore would lead to invasion of the pore’s walls by lipids. In **(C)**, the SNAREs are shown fully zippered at the waist of the pore, but the actual structure is unknown. The fusion pore can fluctuate in size, and flicker open and shut multiple times before expanding further, leading to full fusion **(D)**, or resealing, concluding a transient or kiss-and-run fusion event **(E). (F)** Structure of the *cis*-SNARE complex (adapted from Stein et al., 2009), PDB file 3HD7, rendered in PyMol). The t-SNAREs Syntaxin 1A and SNAP25 are shown in red and salmon, respectively; the v-SNARE Syb2 is shown in blue; TMDs are shown inserted into a membrane. Beta-branched residues are indicated in orange (Stx1) or cyan (Syb2). Note that the ultimate residue in Syb 2 and the last two C-terminal residues in Stx1 were not resolved in the structure and are absent from the image. The plasma membrane thickness is slightly larger than the TMD lengths (Sharpe et al., 2010). **(G)** Alignment of TMD sequences of Syb2 and Stx1 according to the crytal structure shown in **(F)**. Contacts between residues observed in the crystal structure are indicated as black lines. The dashed lines indicate residues that face one another, but are further apart, as the two helices veer apart toward the very C-termini. Beta-branched residues are indicated in orange (Stx1) or cyan (Syb2), as in **(F)** modified from Stein et al. (2009). **(H)** Alignment of TMDs of Syb2 from several species. Uniprot identifiers (http://www.uniprot.org) are indicated in parentheses. The arrows mark the Gly in position 100 (using the rat sequence as reference) and the tiny residue in position 103. See Hastoy et al. (2017) for a more comprehensive alignment.

The neuronal/exocytotic soluble N-ethyl maleimide sensitive factor attachment protein receptors (SNAREs) consist of the v-SNARE Synaptobrevin/VAMP2 (Syb2) and the t-SNAREs Syntaxin-1 (Stx) and SNAP25 (SN25; Sollner et al., 1993). The α-helical SNARE domains of these proteins (highly conserved 60–70 residue cytoplasmic regions) assemble in a parallel coiled coil (with all the N-termini at the membrane-distal end) that brings the membranes to be fused into close proximity (Figure 1; Sutton et al., 1998). It is less clear what happens as the SNARE complex assembly proceeds toward the membrane-proximal ends. The juxtamembrane regions (JMRs) have a propensity to zipper (Gao et al., 2012), with possible functional implications (Stein et al., 2009; Hernandez et al., 2012). These domains are rich in positively charged residues (Neumann and Langosch, 2011) that bind and recruit acidic phospholipids, including PI(4,5)P_2_ (van den Bogaart et al., 2011; Honigmann et al., 2013) and PI(3,4,5)P_3_ (Khuong et al., 2013) to vesicle docking and fusion sites (Barg et al., 2010; Gandasi and Barg, 2014).

Fusion formally occurs once the vesicular lumen is connected to the extracellular space via a fusion pore. Nevertheless, the dynamics of the pore can further control the release process (Breckenridge and Almers, 1987; Zimmerberg et al., 1987; Monck and Fernandez, 1996; Lindau and Alvarez de Toledo, 2003). The pore is initially small (a few nm in diameter) and can flicker open and closed repeatedly before resealing or dilating irreversibly. In transient “kiss and run” fusion, the pore reseals before complete emptying of the vesicle. Alternatively, the pore may dilate irreversibly as the fused vesicle’s membrane collapses into the plasma membrane and the entire cargo is released (full fusion). Thus, beyond affecting the kinetics and the amount of cargo released, pore dynamics also control the mode of vesicle recycling. Transient fusion is a well-established mode of hormone release by neuroendocrine cells (Breckenridge and Almers, 1987; Zimmerberg et al., 1987; Monck and Fernandez, 1996; Lindau and Alvarez de Toledo, 2003; Fulop et al., 2005). It is also documented for synaptic vesicle exocytosis (Staal et al., 2004; He et al., 2006; He and Wu, 2007; Alabi and Tsien, 2013), but its prevalence and significance are debated, in part due to technical challenges in probing fusion pores during neurotransmitter release.

Molecular mechanisms that regulate pore dynamics are not well understood (Lindau and Alvarez de Toledo, 2003; Harata et al., 2006; He and Wu, 2007; Lindau, 2012). Fusion pore properties are affected by calcium (Hartmann and Lindau, 1995; Chiang et al., 2014), dynamin (Anantharam et al., 2011; Chiang et al., 2014), the actin cytoskeleton and/or membrane tension (Bretou et al., 2014; Wen et al., 2016), phosphorylation (Staal et al., 2004), molecular crowding (Wu et al., 2017) and mutations in many of the components of the fusion machinery. In this review article, we emphasize the role of SNARE TMDs in regulating fusion pore dynamics. We choose this focus because SNAREs and Syt are the only TMD proteins known to be involved in the late stages of pore opening and dilation, and few systematic studies of the role of Syt1 TMD in exocytosis are available (Lee and Littleton, 2015). However, we stress that in addition to affecting pore dynamics (Han et al., 2004; Borisovska et al., 2005; Chang et al., 2015, 2016; Dhara et al., 2016), SNARE TMDs also regulate pre-fusion stages (Chang et al., 2016; Dhara et al., 2016). In addition, for nearly all of the proteins mentioned above there are mutations with fusion pore phenotypes (Wang et al., 2001, 2003; Jorgacevski et al., 2011; Dhara et al., 2014). In particular, interfering with synaptotagmin’s calcium binding and/or membrane penetration (Paddock et al., 2011; Lai et al., 2015) influence fusion pore opening and pore properties (Chapman, 2008). Since Synaptotagmin also binds SNAREs (Lai et al., 2014; Zhou et al., 2015), its effects on fusion pores may be difficult to disentangle from those of SNARE protein TMDs. Finally, in addition to possible direct influences, TMDs of other proteins may indirectly influence the fusion pore via their interactions with SNARE TMDs (e.g., the synaptophysin TMD interacts with the Syb2 TMD, Adams et al., 2015).

SNARE TMDs seem to possess some special features. First, compared to other tail-anchored proteins, SNARE TMDs are enriched in beta-branched Ile and Val residues (Neumann and Langosch, 2011). Beta-branched residues (Ile, Val, or Thr) contain two non-hydrogen substituents attached to their C-β carbon, compared to other amino acids that contain only one (Popot and Engelman, 2000). The increased bulkiness near the protein backbone makes it harder for β-branched amino acids to adopt α helical conformations in solution. Nevertheless, such residues are frequently found in α-helical TMDs where they are thought to increase the conformational flexibility of the TMD (Popot and Engelman, 2000). Second, the tiny, helix-perturbing Gly is enriched in the N-terminal portion of the TMD (Neumann and Langosch, 2011) which may allow a kink in the TMD helix (Han et al., 2016b). In comparison, viral fusion protein TMDs are also enriched in Gly (Cleverley and Lenard, 1998), but at a more central position (Neumann and Langosch, 2011). Finally, the TMDs of exocytotic SNAREs are exceptions to the observation that for most proteins the TMD length matches the thickness of membrane wherein the protein resides (Sharpe et al., 2010). Neuronal SNARE TMDs are shorter than the average plasma membrane thickness.

Exocytosis is unique among all biological fusion reactions in that pore dynamics can be observed with sub-millisecond temporal resolution under native conditions using high resolution electrophysiological and electrochemical methods (Travis and Wightman, 1998; Lindau, 2012). In addition, high temporal resolution of single-pore measurements was recently achieved in biochemically defined systems, promising to illuminate many mechanistic questions (Nikolaus and Karatekin, 2016; Stratton et al., 2016; Wu et al., 2016, 2017). Finally, atomistic (Blanchard et al., 2014; Han et al., 2016b) and coarse-grained (CG) simulations (Risselada et al., 2011; Han et al., 2015; Mostafavi et al., 2017) of fusogen protein-membrane systems have provided important insights into the role of TMDs in fusion pore regulation.

## Membrane Fusion Pathways

Fusion between purely lipidic membranes is non-specific and relatively slow (Chanturiya et al., 1997; Warner and O’Shaughnessy, 2012a). In consequence, biological membrane fusion requires specific proteins to perform the recognition and fusion steps. Despite great diversity in biological fusion reactions, from enveloped virus infection, cell-cell fusion, intracellular trafficking and wound repair to exocytosis, the fusogens involved share some general evolutionary principles and drive fusion through a limited number of pathways (Figure 1). First, at some stage the fusogens must be anchored to both of the bilayers. Second, a conformational change in the fusogens brings the hydrated phospholipid head groups into close contact. Fusion requires that substantial hydration forces be overcome (Rand and Parsegian, 1989) and that intermediate high energy states be transiently assumed, in which lipid arrangements are far from that of the equilibrium bilayer. How the associated barrier to fusion is overcome by fusion proteins has been much debated. It was proposed that fusion is triggered when SNARE proteins cooperatively generate entropic forces that clear the contact zone between apposing membranes and push them into close proximity, causing rapid fusion due to thermally driven collisions (Mostafavi et al., 2017). Fusion may be promoted by local destabilization of the bilayer structure, for example by bulging (Martens et al., 2007; Hui et al., 2009). In a radically different possible scenario, the initial fusion pore is a channel-like structure formed by assembly of two hemi-channels in the two fusing membranes (Breckenridge and Almers, 1987; Jackson and Chapman, 2008; Chang et al., 2017; Figure 1B′). In this view the channel somehow subsequently dilates, allowing lipids to invade the pore (Chang et al., 2017).

The fusion pathway may be different in different systems and remains controversial, but in some cases has been shown to pass through or terminate in a hemifused state in which only the proximal leaflets of the apposing membranes are fused while the distal leaflets engage in an extended bilayer region called a hemifusion diaphragm (Figure 1B). These include calcium-mediated fusion of protein-free giant unilamellar vesicles (GUVs; Nikolaus et al., 2010; Warner and O’Shaughnessy, 2012b), fusion of yeast vacuoles (Reese et al., 2005; Jun and Wickner, 2007), and fusion between liposomes mediated by SNAREs alone (Lu et al., 2005; Hernandez et al., 2012) or together with Syt and/or Cpx (Schaub et al., 2006; Diao et al., 2012). Hemifusion was recently observed during exocytosis in chromaffin cells (Zhao et al., 2016).

Truncating SNARE TMDs or replacing them with lipids spanning a single membrane leaflet usually impairs fusion and results in hemifusion (McNew et al., 2000; Xu et al., 2005; Fdez et al., 2010; Chang et al., 2016). Thus TMDs can affect the pathway, either by helping to bypass dead-end hemifusion, or by converting hemifusion to fusion.

## Regulation of Exocytotic Fusion Pores by SNARE TMDs

Once the two membranes have fused, the SNARE complex is now in *cis*, i.e., both the Syb2 and Stx TMDs are embedded in the same membrane. There are several features of the TMDs which may influence pore opening and dynamics at this stage: (1) flexibility of the TMDs; (2) specific interactions between TMDs; and (3) TMD-lipid interactions. These effects are difficult to disentangle. In addition, membrane properties (curvature and tension) and the soluble portions of the fusogens and their interactions with one another and with other proteins will constrain the configurations available to the TMDs.

### TMD Flexibility

Increased flexibility may promote membrane fusion by allowing TMDs to sample conformations compatible with membrane shape changes that accompany fusion (Langosch et al., 2007; Neumann and Langosch, 2011). Consistent with this view, in reconstituted bulk fusion assays fusion activity correlated with β-branched residue content in the TMD sequence (Hofmann et al., 2004; Langosch et al., 2007), although some of these experiments used small sonication-generated liposomes that can be prone to fusion even without fusogens, and leakiness during fusion was not always tested.

TMD flexibility was also identified as a key factor in secretion kinetics in recent studies of exocytosis from mouse chromaffin cells. Dhara et al. (2016) studied cells lacking Syb2 and Cellubrevin (Syb3), that expressed exogenous Syb2 with the entire TMD replaced with a sequence containing various combinations of only Ile, Leu or Val. The results ranged from severe impairment to normal exocytosis. The degree of restoration of secretion correlated well with the fraction of β-branched residues that the Syb2 TMD sequence contained in its N-terminal half (that portion embedded in the cytoplasmic leaflet of the vesicular/plasma membrane). Replacing the wild-type TMD with a polyL stretch reduced the amplitudes of the rapid phases of exocytosis ~5 fold and the slow phase <2 fold as measured by whole-cell capacitance. Interestingly, however, the kinetics were unaffected. In amperometric measurements of single-vesicle release events, replacing the native TMD by polyV or polyI led to shorter, faster rising spikes (∝ amount of catecholamine flux reaching the detector), and shorter, higher amplitude pre-spike features (related to release through the initial pore). Further, pre-spike fluctuations increased in frequency and amplitude. Replacing the TMD by an α-helix-stabilizing polyL sequence produced the opposite effects. These findings suggest that β-branched residues destabilize the pore, facilitating its nucleation (increasing event frequency), and accelerating pore dilation (shorter pre-spike duration and sharper spikes).

In the VAMP2 TMD, a highly conserved Gly100 is followed by another tiny residue three positions later (Cys, Gly, or Ala, Figure 1H), which may be important in allowing a kink in the TMD toward the middle of the bilayer (Han et al., 2016b). Hastoy et al. (2017) studied the role of VAMP2 G100 and C103 by substituting them with Val. These substitutions should impair the ability of the TMD to kink. Using both short peptides encompassing the TMD and full-length purified VAMP2 reconstituted in artificial lipid membranes, the authors found that substitution of C103 and especially of G100 with Val rendered the membranes less fluid and impaired a transition from alpha-helical to beta-sheet conformation of the TMD as the protein concentration increased. These effects correlated with reduced exocytosis in PC12 and INS-1 cells when the knocked-down endogenous VAMP2 expression was rescued with the mutants. Fusion pores expanded faster for the Val mutants, but they also resealed faster after discharging less cargo—a result compatible with transient, kiss and run fusion.

These effects of TMD flexibility on fusion pores are further discussed in the “Computer Simulations of SNARE TMDs and Their Influence on the Fusion Pore” section.

### TMD-TMD Interactions

Interactions among v- and t-SNARE TMDs can take three forms: homotypic (vTMD-vTMD or tTMD-tTMD), heterotypic (vTMD-tTMD) or interactions of SNARE TMDs with TMDs of other proteins, e.g., synaptophysin (Adams et al., 2015).

Homodimerization of v-SNARE TMDs has long been known (Washbourne et al., 1995; Roy et al., 2004; Langosch et al., 2007), but it has been argued that these weak interactions are of little consequence for exocytosis (Bowen et al., 2002; Fdez et al., 2010; Dhara et al., 2016). Neuronal t-SNAREs form clusters in artificial membranes (Bacia et al., 2004; Murray and Tamm, 2009), neuroendocrine cells (Lang et al., 2001; Barg et al., 2010; van den Bogaart et al., 2011; Honigmann et al., 2013, Honigmann-NSMB13; Gandasi and Barg, 2014) and at the neuromuscular junction (Khuong et al., 2013). However, these clusters seem to arise from electrostatic interactions between phospholipids and the JMR of Stx, or from recruitment by vesicle docking (Gandasi and Barg, 2014) rather than specific TMD-TMD interactions.

It has also been proposed that both t- and v-SNARE TMDs may homo-oligomerize into channel-like structures (Han et al., 2004; Jackson and Chapman, 2008; Chang et al., 2015). Remarkably, systematic mutagenesis showed that residues that affected fusion pore currents all fall on one side of the t-SNARE TMD helix, possibly facing the pore’s lumen (Han et al., 2004). In order to release cargo from the vesicular lumen to the extracellular space, a pore lined with t-SNARE TMDs would require a complementary pore formed on the vesicular side by oligomerization of v-SNARE TMDs. Although some evidence supports this idea (Chang et al., 2015; Bao et al., 2016), it is less compelling than that for t-SNARE TMDs. It is also possible that the vesicular hemi-channel includes TMDs from another vesicular protein such as synaptophysin (Chang et al., 2017). A channel-like pore might constitute only the initial structure, yielding a lipid-lined pore once the pore expands (Chang et al., 2017). Since the initial pore lasts only a few milliseconds, it is difficult to confirm or refute such a highly transient, channel-like structure. The notion of a channel-like structure would gain credibility if the contacts between channel-forming units could be identified and manipulated, e.g., to stabilize the pores.

Interactions between v- and t-SNARE TMDs were reported nearly two decades ago (Poirier et al., 1998; Margittai et al., 1999). More recently, Stein et al. (2009) solved the crystal structure of the neuronal SNARE complex including the Syb2 and Stx TMDs in the presence of detergent. The α-helices of Syb2 and Stx1 continued beyond the SNARE domain to the C-termini, spanning the linker region and the TMDs. Contacts between certain Syb2 and Stx1 residues were identified in both linker and TMD domains (Figures 1F,G).

This raises an interesting question: do the TMD-TMD contacts represent specific interactions promoting SNARE complex zippering through the bilayer and affecting the fusion process, or are they artifacts of crystal packing constraints? Wu et al. (2016) sought to answer this question using a novel approach in which single fusion pores can be probed in a biochemically defined system. Three isoleucines in the Syb2 TMD that contact Stx1 TMD residues in the crystal structure were mutated to alanines, and the fusion rate and individual pore properties were monitored. The manipulation reduced the fusion rate moderately, but increased pore lifetimes 10-fold, from ~6 s to ~60 s. Replacing the entire TMD with that of a non-exocytotic v-SNARE or a lipid anchor spanning the entire bilayer resulted in qualitatively similar outcomes. These results suggest that specific interactions between Syb2 and Stx1 TMDs are not essential, but may help fine-tune the fusion reaction.

In a later study, Wu et al. (2017) found that pore dilation does not rely on putative v- and t-SNARE TMD interactions, but rather their results support a dilation mechanism from entropic forces generated by crowding of SNARE complexes at the fusion pore.

### TMD-Lipid Interactions

Clusters formed by neuronal t-SNAREs are cholesterol-dependent in artificial membranes (Bacia et al., 2004; Murray and Tamm, 2009; Milovanovic et al., 2015) and in live neuroendocrine cells (Lang et al., 2001). Unlike raft-associated proteins, t-SNAREs were found to be enriched in cholesterol-poor membrane regions. Milovanovic et al. (2015) showed that the TMD of Stx1 alone is sufficient for cholesterol-dependent clustering and argued that this effect originated in hydrophobic mismatch. Cholesterol-rich membrane regions tend to form thicker liquid-ordered L_o_ domains, whereas cholesterol-poor regions tend to form thinner, liquid-disordered (L_d_) domains. Remarkably, the TMD length of most membrane proteins matches the thickness of the membrane in which they normally reside (Mitra et al., 2004; Sharpe et al., 2010), while neuronal/exocytotic SNAREs appear to be exceptions in that their TMDs are considerably shorter than the thickness of the plasma membrane (Sharpe et al., 2010). This length mismatch may introduce lipid-packing defects that can be minimized if the offending TMDs are clustered (Milovanovic et al., 2015).

Addition of even a single charged residue to the lumenal C-terminus of Syb2 inhibits fusion (Ngatchou et al., 2010). In contrast, Syb2 fused to a pH-sensitive GFP via a flexible linker sequence composed of S and G supports exocytosis (Miesenböck et al., 1998). Ngatchou et al. (2010) interpreted this as evidence that a few of the lumenal Syb2 residues adjacent to the TMD may need to move toward the hydrophobic core of the membrane. This would destabilize the vesicular membrane and help open a fusion pore. D’Agostino et al. (2016) argued that at least during yeast vacuolar fusion this “penetration model” is unlikely to hold.

## Computer Simulations of SNARE TMDs and Their Influence on The Fusion Pore

Computational studies are of particular value to the fields of membrane fusion and exocytosis because experimental characterization of the small, short-lived fusion pore is challenging. These small scale and short-lived features are accessible to computer simulations. The most detailed approaches are atomistic, currently accessing up to microsecond timescales (Han et al., 2016b), while CG methods can probe considerably larger times (Cooke et al., 2005; Marrink et al., 2007; Monticelli et al., 2008; Mostafavi et al., 2017).

### TMD Flexibility

Conformational flexibility of the TMDs has been proposed to play a role in fusion (Langosch et al., 2007; Stelzer et al., 2008). Atomistic simulations of a v-SNARE C-terminal fragment in a membrane identified three types of flexibility possessed by the α-helical linker-TMD regions: (i) tilt relative to the membrane normal; (ii) a kink feature at the Gly100 residue; and (iii) conformational flexibility of the entire backbone (Blanchard et al., 2014; Han et al., 2016b). The tilt and kink angles were uncorrelated, as expected for a flexible TMD, yet confined to a narrow range ~10^0^ (Blanchard et al., 2014). Similar kinked conformations were seen in simulations with CG representations of TMDs and lipids (Durrieu et al., 2009; Lindau et al., 2012), and in an atomistic study of a t-SNARE C-terminal fragment (Knecht and Grubmuller, 2003).

Taken together, simulations and experiments suggest that TMD conformational flexibility (in particular kinking and/or backbone flexibility—types (ii) and (iii) above) promotes exocytosis. In chromaffin cell experiments with the Syb2 native TMD replaced by sequences containing only Val, Ile, or Leu, β-branched residue content correlated with restoration of secretion (Dhara et al., 2016). Simulations appear to identify flexibility as the relevant property, because polyI and polyV substitutions increased simulated backbone TMD flexibility, while polyL substitution decreased the flexibility (Han et al., 2016b). By contrast, in these simulations all three substitutions reduced the tilt and straightened the TMD compared to wildtype, while fluctuations in the associated angles were reduced. These results suggest tilt and kink flexibilities are of minor importance to pore dilation. However, mutations of the Syb2 residues Gly100 and Cys103 to Val that would be expected to reduce the N-terminal kinking resulted in a decrease in exocytosis and pores that expanded and closed faster following partial release (Hastoy et al., 2017). Therefore, both types of flexibility (kinking and backbone fluctuations) appear to be relevant.

Thus, increased TMD flexibility may favor pore nucleation and expansion. What is the underlying mechanism? Greater TMD flexibility in the N-terminal portion of the TMD might splay lipids and so relieve the high negative curvature in the outer phospholipid leaflet in a small fusion pore, thereby promoting pore nucleation (Dhara et al., 2016). Dhara et al. (2016) also proposed that this mechanism would promote pore expansion, but this is less clear since pore geometry is complex. Consistent with the ability of TMDs to moderate lipid ordering, TMDs disturbed lipid ordering in their vicinity in atomistic and Martini simulations (Risselada et al., 2011; Han et al., 2016b). TMD flexibility may also promote fusion more directly, by helping membranes to assume configurations required to navigate pathways to fusion. Further, several known or potential TMD-TMD interactions may be affected by Syb2 TMD mutations. One might expect that any specific interactions would be disrupted by replacing the TMD with a simple I, L, or V repeat sequence (Dhara et al., 2016). However, specificity in TMD-TMD interactions may be encoded in just a single TMD residue in a cellular context, likely due to packing interactions (Heim et al., 2015).

Many questions remain unanswered regarding the role of TMD flexibility in fusion. For example, if indeed greater flexibility translates to more frequent and faster exocytosis, one might anticipate that lipid-anchored Syb2 would provide the most efficient fusion. However, no such enhancement is seen when Syb2 is anchored by palmitoylated cysteine string protein (CSP) in chromaffin cells (Chang et al., 2016; Dhara et al., 2016), neurons (Chang et al., 2016), or when a lipid anchor that spans the entire bilayer is used (McNew et al., 2000; Wu et al., 2016, 2017).

A mathematical model suggested that another possible mechanism whereby TMD flexibility could promote fusion is by enhancing entropic forces tending to expand the pore. In the model, increased TMD flexibility would increase orientational fluctuations of and mutual steric interactions among the cis-SNARE complexes, increasing the entropic pore expansion force (Wu et al., 2017).

### TMD-TMD Interactions

The mathematical model of Wu et al. (2017) identified a critical role for specific and non-specific TMD-TMD interactions in fusion. These interactions drive zippering of cis-SNARE complexes to the fusion pore waist, forcing the SNAREs to interact sterically and thus generating entropic forces that drive pore expansion.

TMD interactions may be important at other stages of fusion. Simulations show v-SNARE TMDs interact and can homodimerize or form higher order oligomers (Fleming and Engelman, 2001; Han et al., 2015, 2016a), with an interaction energy of ~10 *k*_B_*T* between v-SNARE C-terminal fragments measured in a hybrid atomistic-MARTINI approach (Han et al., 2015). A MARTINI study suggested that the fusion pathway passes through a hemifused state with a HD, and that homodimerization of SNARE TMDs restricts the HD to remain small and therefore to transit more readily to a fusion pore (Risselada et al., 2011).

### TMD-Lipid Interactions

Interactions between lipids and SNARE TMDs or JMRs may assist fusion. In Martini simulations, post fusion SNARE complexes surrounding the fusion pore were constrained to retain their Y shape by the energy penalty associated with moving the C-terminal polar residues through the hydrophobic membrane core (Risselada et al., 2011). Thus, the bending energy stored in the C-terminal portion of the complexes could be released only by pore expansion. Other MARTINI and hybrid atomistic/CG studies have shown that PI(4,5)P_2_ concentrates at t-SNARE JMRs due to interactions with the charged Lys and Arg residues (Khelashvili et al., 2012; Sharma et al., 2015). These effects are thought to help cluster neuronal t-SNAREs (van den Bogaart et al., 2011).

## Author Contributions

ZW, ST, BO’S and EK contributed to the review of the literature, and to the writing and editing of the manuscript. ZW and EK produced the initial draft. EK coordinated the work.

## Conflict of Interest Statement

The authors declare that the research was conducted in the absence of any commercial or financial relationships that could be construed as a potential conflict of interest.
